# Functional and Psychobiotic Potential of a Food-Derived Multi-Strain Lactic Acid Bacteria Consortium: An In Vitro Evaluation Using Static Digestion and SHIME^®^ Models

**DOI:** 10.3390/nu18121946

**Published:** 2026-06-16

**Authors:** Wioletta Mosiej, Marcin Kruk, Tomasz Królikowski, Michał Oczkowski, Klaudia Glegoła, Dorota Zielińska

**Affiliations:** Institute of Human Nutrition Sciences, Warsaw University of Life Sciences—SGGW, Nowoursynowska 159c, 02-776 Warsaw, Poland; wioletta_mosiej@sggw.edu.pl (W.M.); marcin_kruk@sggw.edu.pl (M.K.); tomasz_krolikowski@sggw.edu.pl (T.K.); michal_oczkowski@sggw.edu.pl (M.O.); s210375@sggw.edu.pl (K.G.)

**Keywords:** food-derived LAB, microbiota–gut–brain axis, multi-strain, in vitro, SHIME, psychobiotic

## Abstract

Background/Objectives: The microbiota–gut–brain axis (MGBA) plays a pivotal role in cognitive function, making psychobiotics a promising strategy for managing neurodegenerative diseases. Lactic acid bacteria (LAB) from traditional fermented foods represent a valuable source of candidate strains, and multi-strain consortia may offer enhanced therapeutic efficacy through synergistic effects. This study evaluated the functional and psychobiotic potential of three lactic acid bacteria (LAB) strains isolated from fermented foods, assessed as monocultures and a multi-strain consortium (MIX). Methods: The research encompassed an initial screening of the individual strains and the MIX, assessing their adhesion to mucin, stability in a static in vitro digestion model, and amino acid profiling. Subsequently, the LAB MIX underwent long-term evaluation in a dynamic gastrointestinal model (SHIME^®^) inoculated with microbiota from a patient with Alzheimer’s disease, during which alterations in gut microbiota composition and amino acid metabolism were analyzed. Results: The LAB MIX demonstrated high stability under digestive stress and effective mucoadhesive properties. Furthermore, the consortium demonstrated a distinct metabolic signature, driving enhanced functional effects that complemented or exceeded those observed in individual monocultures. In the SHIME^®^ model, the MIX induced significant, site-specific shifts in microbial composition, notably increasing lactobacilli abundance. These taxonomic changes correlated with an enriched metabolic profile, including elevated levels of GABA precursors and amino acids with antioxidant potential, which are crucial for MGBA modulation. Conclusions: These results identify the LAB consortium as a compelling psychobiotic candidate. Further in-depth in vivo and clinical studies are required to validate its therapeutic potential for MGBA modulation.

## 1. Introduction

The gut–brain axis (also referred to as microbiota–gut–brain axis, or MGBA) is a bidirectional communication network connecting the gastrointestinal tract (GIT), its microbiota, and the central nervous system through neural, immune, endocrine, and metabolic pathways [1,2]. Growing evidence indicates that the microbiome has a significant impact on mental health, cognition, emotion regulation, synaptic plasticity, and neurodevelopment [3]. The gut microbiota (GM) profoundly influences the central nervous system by producing a wide range of bioactive metabolites, including short-chain fatty acids (SCFAs), amino acid derivatives, and neurotransmitters. These compounds modulate brain function either directly, by crossing the blood–brain barrier, or indirectly, by activating afferent enteric nerves through intestinal epithelial and enteroendocrine cell receptors [4,5]. Consequently, dysbiosis, defined as an imbalance in microbial composition and diversity, can disrupt these diverse metabolic and immunomodulatory pathways. This disruption has been associated with psychiatric disorders such as anxiety, depression, and schizophrenia, as well as neurodevelopmental conditions such as autism spectrum disorder and neurodegenerative disorders, including mild cognitive impairment, Parkinson’s disease, and Alzheimer’s disease (AD) [6,7].

The rising global burden of mental health and neurodegenerative disorders, along with the limitations and adverse effects of conventional pharmacotherapies, has sparked growing interest in microbiome-targeted interventions. Beyond conventional treatment strategies, approaches targeting the MGBA, such as psychobiotics, offer a promising, potentially safer, and cost-effective complementary strategy to support brain health [8]. Psychobiotics, broadly defined as live microorganisms or microbe-derived products that interact with the MGBA, are recognized for their neuroactive potential mediated through integrated neural, immune, endocrine, and metabolic pathways [9,10]. Accordingly, interventions targeting these metabolic and anti-inflammatory mechanisms highlight the emerging potential of lactic acid bacteria (LAB) derived from traditional fermented foods, which may also represent an important reservoir of psychobiotic potential. Their robust survival under acidic and bile environments facilitates gastrointestinal transit, positioning these food-origin microorganisms as a promising and accessible means to modulate the MGBA and support neurological health [11,12].

Although much research has traditionally focused on the properties of isolated single strains, growing evidence suggests that multi-strain consortia may offer superior therapeutic outcomes through functional synergy and additive effects. Unlike monoculture interventions, these microbial mixtures may offer greater benefits than the same strains used individually [13,14]. Consequently, investigating the complementary potential of combined food-derived LAB strains represents a critical step toward developing more effective, broad-spectrum therapeutic strategies. Considering the widespread depletion of the gut microbiome in Western populations and the markedly reduced consumption of fermented foods compared with previous generations, dietary supplementation with selected bacterial consortia isolated from fermented foods may represent a promising strategy to support modern approaches to the prevention and treatment of lifestyle-related diseases [15].

This study aimed to evaluate and compare the functional and psychobiotic potential of three food-derived LAB strains, both as individual isolates and as a combined multi-strain formulation, by assessing adhesion to mucin, amino acid metabolic profiles, and bacterial viability in a static gastrointestinal digestion model. Furthermore, to validate the stability and fermentative activity of the microbial mixture under physiologically relevant conditions, the consortium was subjected to long-term assessment using the Simulator of the Human Intestinal Microbial Ecosystem (SHIME^®^), a dynamic in vitro model of the human gut.

## 2. Materials and Methods

### 2.1. Experimental Design

The research was conducted in two consecutive phases to evaluate the functional properties and potential complementary effects of three food-derived LAB strains, tested both as individual monocultures and as a multi-strain consortium (MIX).

(1) In the first phase, a comparative screening was conducted to characterize the strains and their mixture, focusing on their mucin-adhesion capacity and stability during static in vitro GI digestion. Additionally, the amino acid profiles of samples were quantified to establish a metabolic baseline.
(2) In the second phase, following the preliminary screening, the bacterial consortium (MIX) was selected for advanced validation using the Simulator of the Human Intestinal Microbial Ecosystem (SHIME). This stage focused on monitoring the consortium’s impact on GM composition and evaluating subsequent shifts in amino acid metabolism under realistic, simulated colonic conditions.

### 2.2. Bacteria Preparation

Three LAB strains, *Lactiplantibacillus plantarum* KY744440.1 (OS4), *Levilactobacillus brevis* GCF_040112745.1 (O24), and *Lactiplantibacillus pentosus* PP864640 (M3.3), isolated from traditional Polish fermented foods and sourced from the Warsaw University of Life Sciences (Department of Food Gastronomy and Food Hygiene) collection, were utilized in this study. The strains were previously characterized regarding their basic properties and selected based on their immunological and antioxidant activity [12]. Strains were revived from −80 °C storage on MRS agar (Merck, Darmstadt, Germany) and incubated anaerobically at 37 °C for 24 h. A single colony was then transferred to MRS broth and cultured for an additional 24 h. Following a final passage, overnight cultures in the early stationary phase were used for the experiments. To ensure uniformity, all individual strains were harvested by centrifugation, washed in sterile phosphate-buffered saline (PBS, Sigma-Aldrich, St. Louis, MO, USA), and initially adjusted to a standardized concentration of 10^9^ CFU/mL. The ‘LAB MIX’ was prepared by combining the standardized individual strains in an equal volume ratio (1:1:1 based on CFU). The consortium was then resuspended in either MRS broth or SHIME basal medium to achieve a final concentration of approximately 3.3 × 10^8^ CFU/mL for each strain. This maintained a total concentration of 10^9^ CFU/mL for the final MIX, thereby ensuring a standardized inoculum for subsequent experiments.

### 2.3. Static In Vitro Digestion

The static in vitro digestion was conducted according to the INFOGEST 2.0 [16] standardized guidelines, comprising oral, gastric, and intestinal phases. Simulated salivary (SSF), gastric (SGF), and intestinal (SIF) fluids were prepared as 1.25× electrolyte stock solutions, with pH adjusted to 7.0, 3.0, and 7.0, respectively, using 5 M HCl and 5 M NaOH (Chempur, Piekary Śląskie, Poland). The digestive process was carried out at 37 °C with continuous stirring, utilizing specific enzymatic activities for each stage: 75 U/mL α-amylase (from Aspergillus oryzae, Sigma-Aldrich, St. Louis, MO, USA; ~30 U/mg) for the oral phase, 2000 U/mL pepsin (from porcine gastric mucosa, Sigma-Aldrich, St. Louis, MO, USA; ~2500 U/mg) for the gastric phase, and a combination of 100 U/mL pancreatin (from porcine pancreas, Sigma-Aldrich, St. Louis, MO, USA; ~6.5 U/mg) and 10 mM bovine bile salts (Sigma-Aldrich, St. Louis, MO, USA) for the intestinal phase. All solutions were preheated to 37 °C before use. The specific volumes, mixing ratios, and incubation times for each digestion stage are summarized in Table 1.

Samples (20 µL) were collected at baseline (prior to the commencement of the simulated digestion) and after the gastric and intestinal phases, then transferred to 180 µL of sterile PBS (Sigma-Aldrich, St. Louis, MO, USA). Bacterial survival was determined by 10-fold serial microdilutions in 96-well plates and subsequent plating on MRS agar (Oxoid, Basingstoke, UK). Following a 48 h anaerobic incubation at 37 °C (generated using AnaeroGen bags, Thermo Scientific, Basingstoke, UK), colonies were quantified and expressed as CFU/mL. The experiment was performed in three biological replicates.

### 2.4. Mucin Adhesion Assay

Dry, powdered porcine stomach mucin (Type-III, Sigma-Aldrich, St. Louis, MO, USA) was sterilized by mixing it with anhydrous EtOH in a sterile Falcon tube. Then, the EtOH–mucin suspension was poured into a sterile Petri dish and dried overnight at 50 °C. After drying, mucin was resuspended in sterile PBS (Sigma-Aldrich, St. Louis, MO, USA) to a final concentration of 5 mg/mL. Then, 200 µL of PBS–mucin solution was portioned into each well of 96-well microtitre plates, and incubated for 24 h at 4 °C for mucin coating. After that time, the excess mucin solution was removed, and the plates were gently washed once with sterile PBS. Into each well, 180 µL of MRS media was portioned. The OD600 of overnight LAB isolate cultures was normalized to 0.6 ± 0.1 using a SpektraMax iD3 (Molecular Devices, San Jose, CA, USA), which corresponds to 7.5 ± 0.2 log CFU/mL. Then, 20 µL of normalized culture was used to inoculate a mucin-coated plate with MRS media. Cultures were incubated for 12 h at 37 °C. After incubation, the bacterial suspension was removed, and the wells were washed 3 times each with 200 µL of sterile PBS, then dried for 30 min at 70 °C in a laboratory oven. To quantify the amount of LAB that adhered to the mucin coating during incubation, 200 μL of 0.1% crystal violet solution in Milli-Q water was poured into each well. After staining for 30 min, the solution was removed. The wells were then gently washed three times with 200 µL of Milli-Q water. Next, the plates were dried in a laboratory oven at 60 °C for 30 min, or until all moisture evaporated. Then, 200 µL of 33% acetic acid was added to each well to extract crystal violet for 40 min. Subsequently, 150 µL of the crystal violet-acetic acid solution per well was transferred to a new 96-well plate. OD was measured using a SpectraMax iD3 at 570 nm. The experiment was performed in three biological replicates.

### 2.5. Free Amino Acids

Amino acids were determined using a method adapted from Redruello et al. [17]. Reagents and standards were purchased from Sigma-Aldrich (Poznań, Poland). Before analysis, samples were centrifuged at 10,000× *g* for 10 min at 15 °C using an Eppendorf 5804 R centrifuge equipped with an FA-45-48-11 rotor (Hamburg, Germany). The obtained supernatants were subsequently ultrafiltered using 3 kDa cut-off Amicon filters (Merck Millipore, Burlington, MA, USA) and centrifuged for 30 min at 10,000× *g* at 15 °C. The resulting supernatants were mixed (1:1, *v*/*v*) with 0.1 N HCl containing 0.2% (*w*/*v*) 3,3′-thiodipropionic acid (TDPA). Stock solutions (10 mM) of amino acids were prepared in 0.1 N HCl containing 0.2% (*w*/*v*) TDPA, and working standards were obtained by appropriate dilution in the same solution, including L-2-aminoadipic acid as an internal standard. For derivatization, 100 μL of sample or standard solution was mixed with 175 μL of 1 M borate buffer (pH 9.0), 75 μL of methanol, 2 μL of internal standard solution (1.9 g/L), and 3 μL of diethyl ethoxymethylenemalonate (DEEMM). The mixture was incubated in an ultrasonic bath at 30 °C for 45 min and subsequently heated at 70 °C for 2 h to degrade excess reagent and by-products. The samples were filtered through 0.22 μm membranes before analysis. Chromatographic separation was performed using a Shimadzu HPLC system (Kyoto, Japan) consisting of an LC-20CE pump, CBM-20A controller, SIL-20AC autosampler, CTD-20AC column oven, and SPD-20AV UV/Vis detector (Kyoto, Japan), with detection at 280 nm. Separation was achieved on a Phenomenex Luna Omega Polar C18 column (Torrance, CA, USA; 3 μm, 100 Å, 150 × 4.6 mm) maintained at 35 °C. The mobile phase consisted of (A) 25 mM acetate buffer (pH 6.25) containing 0.02% sodium azide and (B) acetonitrile with 2% methanol, delivered at a flow rate of 1.3 mL/min with an injection volume of 0.5 μL, while the autosampler was kept at 10 °C. During chromatographic separation, the proportion of mobile phase B changed as follows: 0.01–1.20 min, 10%; 1.21–1.90 min, 8%; 2.00–4.00 min, 10–16%; 4.00–7.30 min, 16%; 7.30–9.15 min, 16–29%; 9.15–9.75 min, 29–22%; 9.75–13.25 min, 22–60%; 13.25–13.75 min, 60–82%; 13.75–14.10 min, 82–100%; 14.10–16.00 min, 100%; 16.00–16.25 min, 100–10%, followed by re-equilibration to 20.00 min. Amino acids were identified based on retention times and UV spectra recorded at 280 nm. Quantification was performed using the internal standard method, based on calibration curves prepared from the peak-area ratios of each amino acid to L-2-aminoadipic acid. This internal standard was added at a constant concentration to all calibration standards and samples, and was selected because it is not naturally present in the studied matrices. The experiment was performed in three biological replicates.

### 2.6. Dynamic Gut Microbiota Simulation

#### 2.6.1. Study Design and Inoculum Preparation

Colonic fermentation was simulated using the Simulator of the Human Intestinal Microbial Ecosystem (SHIME^®^; ProDigest, Ghent, Belgium), inoculated with fecal microbiota from a 92-year-old female donor diagnosed with probable AD according to the NINCDS-ADRDA criteria. The donor met strict inclusion criteria, including no antibiotic or probiotic therapy for three months and no history of chronic gastrointestinal or metabolic diseases (Ethics Committee approval no. 24/RKE/2023). The fecal inoculum was prepared in accordance with the manufacturer’s (ProDigest) SHIME operation manual. Briefly, the fresh fecal sample was processed anaerobically within two hours, homogenized (20% *w*/*v* in anaerobic phosphate buffer, pH = 6.9 ± 0.1; ORP was kept below −200 mV before inoculation), and centrifuged at 500× *g* for 2 min (Eppendorf Centrifuge 5804 R, Hamburg, Germany). The resulting supernatant was used to inoculate the bioreactors (5 mL per 100 mL of medium), followed by an overnight stabilization phase.

#### 2.6.2. TWINSHIME Setup

**Figure 1:**
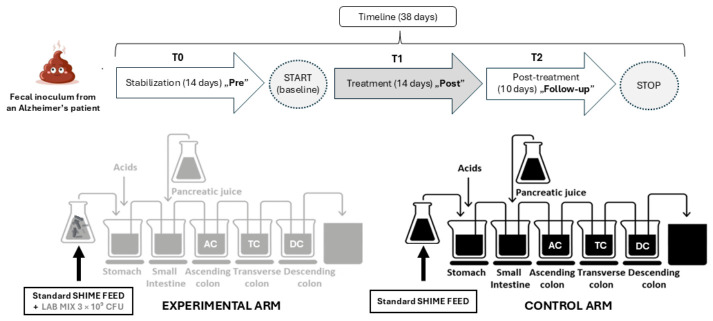
Timeline and schematic overview of the TWINSHIME experiment. TWINSHIME was used to simulate gastrointestinal passage in two parallel lines inoculated with fecal microbiota from an Alzheimer’s disease (AD) donor. After a 14-day stabilization (“Pre”/T0), the experimental arms received a food-derived LAB MIX for 14 days (“Post”/T1), while control arms received only basal medium, followed by a 10-day washout (“Follow-up”/T2). Samples from the ascending colon (AC), transverse colon (TC), and descending colon (DC) were collected at each phase for microbiota and metabolite analyses. On the timeline, large block arrows indicate successive experimental phases, while dashed circles mark the baseline initiation (“START”) and experiment termination (“STOP”). In the reactor schematics, vertical and connecting arrows denote the direction of feed/fluid introduction and the continuous flow of luminal content through the gastrointestinal compartments, respectively.

The TWINSHIME system was used to enable parallel simulation of colonic fermentation in experimental and control arms under identical conditions (Figure 1). Each arm consisted of five double-jacketed bioreactors, representing the stomach (ST), small intestine (SI), and the ascending (AC), transverse (TC), and descending colon (DC), maintained at 37 °C under continuous anaerobic conditions via nitrogen flushing. The pH was automatically monitored and adjusted to physiological levels (ST 2.0; SI 6.6; AC 5.7–5.9; TC 6.15–6.4; DC 6.6–6.9) using 0.5 M HCl and NaOH.

The system followed a standardized feeding regime where 140 mL of basal medium (Feed, PD-NM002B, Prodigest, Ghent, Belgium) was supplied to the ST three times daily, followed by transfer to the SI with 60 mL of pancreatic juice containing NaHCO_3_ (12.5 g/L) (Chem-lab, Zedelgem, Belgium), Oxgall bile (6.0 g/L) (Difco, Detroit, MI, USA), and porcine pancreatin (0.9 g/L) (Sigma-Aldrich, St. Louis, MO, USA). The 38-day study protocol comprised a 14-day stabilization period to establish the fecal microbiota (confirmed by stable SCFA profiles), followed by a 14-day intervention phase during which the LAB MIX was administered once daily with feed to the experimental arm, while the control arm received only the basal medium. Finally, a 10-day washout phase was conducted to assess the persistence of the intervention effects. Colonic samples from the AC, TC, and DC were collected on days 14 (Pre), 28 (Post), and 38 (Follow-up) for microbial and metabolic profiling and stored at −80 °C for further analysis.

#### 2.6.3. Microbiota Analysis, Metabolic Profiling, and Data Processing

Total genomic DNA was extracted from in vitro simulated colonic samples using the Genomic Mini AX Stool (mod.1) kit (A&A Biotechnology, Gdynia, Poland, cat. no. 065-60-M1), following the manufacturer’s protocol. After isolation, DNA quality was checked by running the sample on 1% agarose gel, and template quantity was measured by fluorimetry using Qubit 2.0 and High Sensitivity Picogreen reagents (Thermo Scientific, Waltham, MA, USA). Amplification of conserved bacterial 16S rRNA gene fragment covering V3 and V4 regions was performed in triplicate with the use of the gene-specific primers [18]: 16S_V3-F 341-357F: 5′ TCGTCGGCAGCGTCAGATGTGTATAAGAGACAGCCTACGGGNGGCWGCAG 3′ and 16S_V4-R 785-805R: 5′ GTCTCGTGGGCTCGGAGATGTGTATAAGAGACAGGAC TACHVGGGTATCTAATCC 3′. Obtained amplicons of the size c.a. 450 bp were checked on 1% agarose gel and purified by Ampure XP magnetic beads (Beckman, Brea, CA, USA). Amplicon libraries were pooled in equimolar ratios and indexed according to the Nextera indexing strategy by PCR (Illumina, San Diego, CA, USA). Sample indexing allowed pooling of amplicons for a sequencing run and further extraction of sample sequence reads from a large batch of sequencing results. 16S amplicons were sequenced on the MiSeq sequencer in the DNA Sequencing and Oligonucleotide Synthesis Laboratory IBB PAS in paired-end mode using a 600-cycle v3 chemistry kit (Illumina, San Diego, CA, USA). The amino acid sample profiling procedure for the SHIME experiment samples was performed as described in Section 2.5. Free Amino Acids subsection. All the analyses were performed in three technical replicates.

### 2.7. Statistical Analyses

Statistical analyses were performed in RStudio (version 4.3.2, R Foundation for Statistical Computing, Vienna, Austria). Depending on the comparison, statistical significance was evaluated using Student’s *t*-test or one-way ANOVA followed by Tukey’s or Fisher’s LSD post hoc test, after verifying assumptions of normality and homogeneity of variance. Sequencing data were rarefied to a uniform depth to account for differences in sequencing effort across samples using the phyloseq package. Alpha diversity was assessed using Chao1 richness and Shannon index. Estimates were derived using generalized linear mixed-effects models to account for repeated measures across colon segments and timepoints. Taxonomic classifications were aggregated and converted to relative abundance (percentage contribution of each genus within a sample). For visualization purposes, low-abundance families (≤2.0%) were grouped into a collective category. Changes in microbiota composition were illustrated using bar plots, stratified by colon segment, time point, and experimental group. Figures were generated with ggplot2 (RStudio). Free amino acid concentrations were compared to baseline (T0) within each series for volcano plot visualization. Microbiota data were transformed using the centered-log-ratio after pseudocount (1 × 10^6^) adjustment, while amino acid data were log-transformed and Z-score standardized. The datasets were merged by sample identity. The Principal Component Analysis (PCA) was performed on mean-centered, variance-scaled variables, excluding features with zero variance. Results were visualized using biplots of sample scores and variable loadings.

## 3. Results

### 3.1. Functional Properties of Individual Monocultures and a Multi-Strain Consortium (MIX)—A Comparative Screening

**Figure 2:**
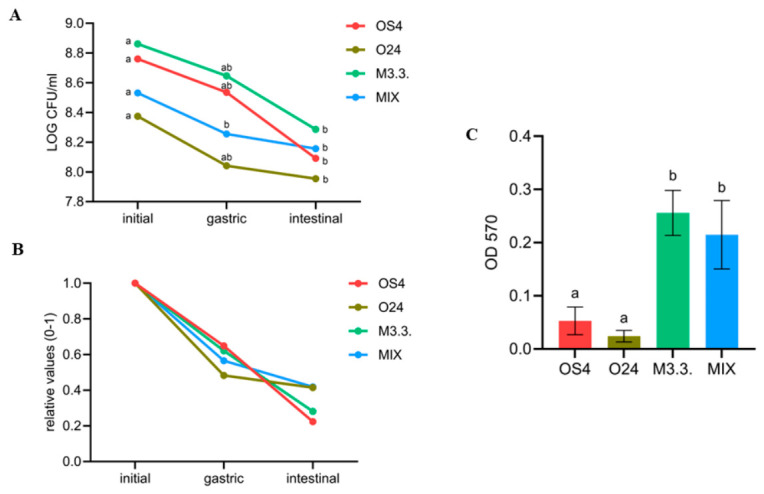
(**A**) Bacteria’s survival during initial (baseline), gastric and intestinal digestion: content in log CFU/mL and (**B**) in relative values (0–1) (Y); lowercase letters indicate statistical differences between samples in Tukey’s test after ANOVA analysis (*p* < 0.05); *n* = 3. (**C**) Adhesion to mucin of the tested bacterial strains measured by spectrophotometric analysis at a wavelength of 570 nm; the absorbance values obtained were proportional to the amount of biofilm formed; lowercase letters indicate statistical differences between samples in Tukey’s test after ANOVA analysis (*p* < 0.05); error bars indicate standard deviations.

All samples exhibited a mild reduction in bacterial counts during simulated digestion (Figure 2A). MIX demonstrated the greatest stability, with the smallest decrease between stages (no sharp decline in bacterial numbers was observed), whereas OS4 and M3.3. showed the most pronounced decline (Figure 2B), particularly in the intestinal phase. However, M3.3. maintained the highest final bacterial concentration (8.27 ± 0.09 log CFU/mL).

Adhesive ability was assessed using a mucin adhesion assay. The highest mean values were obtained for M3.3. (0.26 ± 0.04) and MIX (0.21 ± 0.06); strains showed the best adhesion to mucin, with MIX showing the greatest variability (highest SD) (Figure 2C). The OS4 (0.05 ± 0.03) and O24 (0.02 ± 0.01) strains showed lower mean values and less variation.

**Figure 3:**
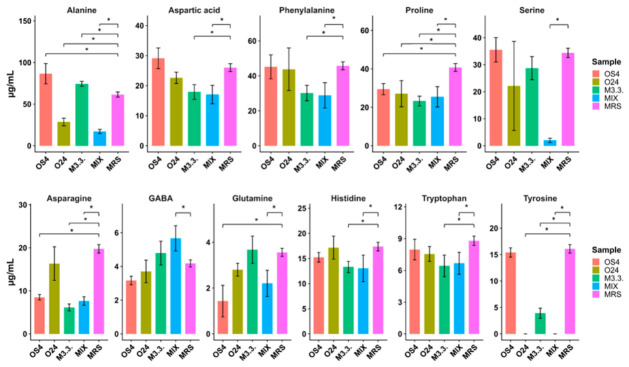
Free amino acid profiles in post-culture fluids of the tested bacterial strains, the multi-strain consortium (MIX), and the MRS control medium. Only amino acids for which statistically significant differences were observed are shown. Statistical significance is indicated as asterisks (*) between the samples and the control medium according to Student’s *t*-test (*p* < 0.05; *n* = 3); error bars indicate standard deviations.

Free amino acid concentrations measured in post-culture fluids differed across the tested strains and MIX (Figure 3). Their metabolic activity significantly altered amino acid profiles relative to the MRS control medium. These effects were most pronounced in the MIX and M3.3. samples, whereas other monocultures exhibited varying degrees of metabolic divergence. The M3.3. showed the closest metabolic affinity to MIX by significantly reducing levels of aspartic acid, phenylalanine, proline, asparagine, histidine, tryptophan, and tyrosine. However, unlike the MIX, M3.3. maintained a higher concentration of alanine. Notably, the MIX formulation was the only sample to significantly deplete serine levels and, conversely, the only one from all tested samples to significantly increase GABA concentration (*p* < 0.05). Notably, tyrosine was completely depleted in both the O24 and MIX samples, while a significant reduction in its concentration was also observed for the M3.3. monoculture compared to the MRS control. Conversely, the OS4 and O24 monocultures showed the least significant divergence from the MRS control, with O24 also exhibiting the highest standard deviation among all tested samples.

**Figure 4:**
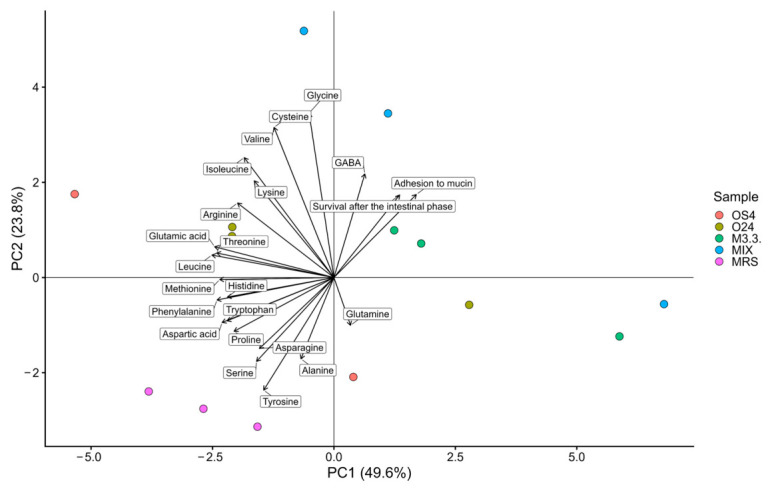
Principal Component Analysis (PCA) biplot illustrating the distribution of samples based on their functional properties. The analysis reveals the correlation between the survival after the intestinal phase, adhesion to mucin and analyzed amino acids (vectors) and the samples (points; *n* = 3). The percentages on the axes indicate the variance explained by each principal component.

Additionally, PCA revealed clear functional feature differentiation among the samples, with the first two principal components explaining 73.4% of the total variance (PC1: 49.6%; PC2: 23.8%) (Figure 4). The samples were distributed across all four quadrants, reflecting distinct survival after the intestinal phase, adhesion to mucin and amino acid profiles. The MRS control, OS4, and O24 samples clustered mostly in the lower-left and upper-left quadrants, showing a strong association with primary amino acids, including aromatic amino acids (tryptophan, phenylalanine, tyrosine). The M3.3. occupied intermediate positions along the PC1 axis, indicating strong correlations with survival after simulated GI conditions and the ability to adhere to mucus, as well as a transitional metabolic state. In contrast, the MIX samples were predominantly located in the upper-right quadrant, exhibiting strong positive correlations with the vectors for gamma-aminobutyric acid (GABA), glycine, and cysteine, and a close association with branched-chain amino acids (BCAAs), specifically valine, isoleucine, and leucine, as well as survival after the intestinal phase and adhesion to mucus.

### 3.2. Microbiota and Metabolic Changes During a Multi-Strain Consortium (LAB MIX) Intervention in the Dynamic Digestive Tract SHIME Model Inoculated with the Microbiota of an AD Patient

**Figure 5:**
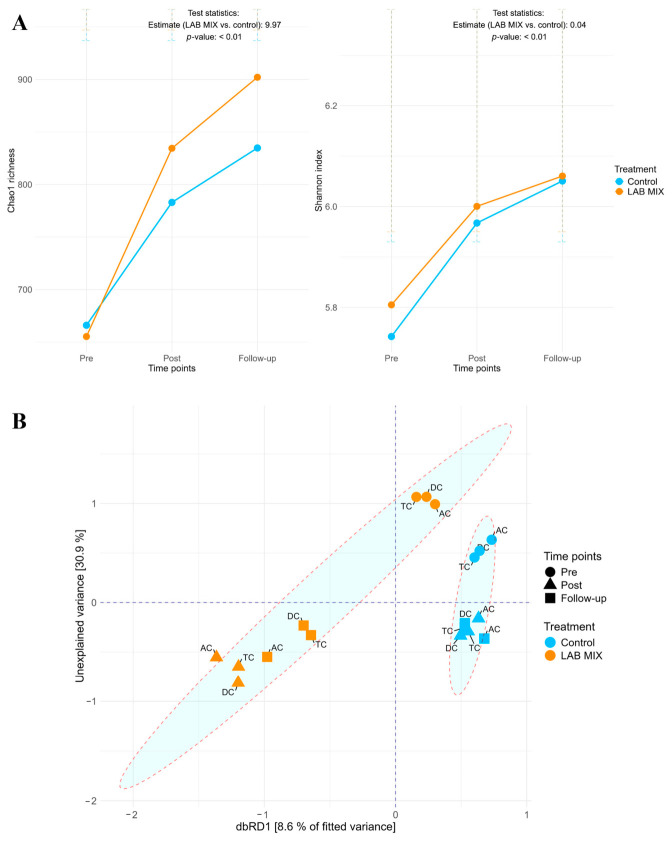
(**A**) Changes in microbial community alpha diversity, measured by Chao1 richness (**left**) and Shannon index (**right**), for the control and LAB MIX treatment groups across time points (Pre, Post, Follow-up). (**B**) Distance-based redundancy analysis (dbRDA) plot based on Bray–Curtis dissimilarity of log-transformed genus-level data, showing the separation between LAB MIX and control groups across time points (Pre, Post, Follow-up) in three colon compartments (AC, TC, DC); ellipses represent 90% confidence intervals, separating the control group (right ellipse) from the LAB MIX treatment group (left ellipse).

Microbial alpha diversity, assessed by both Chao1 richness and Shannon index, showed a consistent upward trend from Pre- to Follow-up time points in both study groups (Figure 5A). Compared to the control, the treatment arm receiving the LAB MIX supplementation exhibited a significantly greater increase in community richness, as evidenced by the Chao1 index (*p* < 0.01). A parallel, statistically significant improvement in microbial diversity was also observed in the LAB MIX arm compared to the control, as measured by the Shannon index (*p* < 0.01). Distance-based redundancy analysis (dbRDA) was used to characterize differences in microbial community structure between the treatment and control groups (Figure 5B). The ordination plot shows a clear separation between the LAB MIX and the control groups along the primary constraint axis (dbRD1). While samples from different time points (Pre, Post, Follow-up) show some overlap, the clustering pattern indicates that the LAB MIX treatment induced a distinct shift in microbial composition compared to the control group. The separation of these two clusters, supported by the constrained variance (8.6%, permutation test, *p* < 0.01), indicates that the LAB MIX intervention exerts a statistically significant, though quantitatively modest, influence on the microbial community structure, with the remaining variance likely driven by temporal and segment-specific factors within the model.

**Figure 6:**
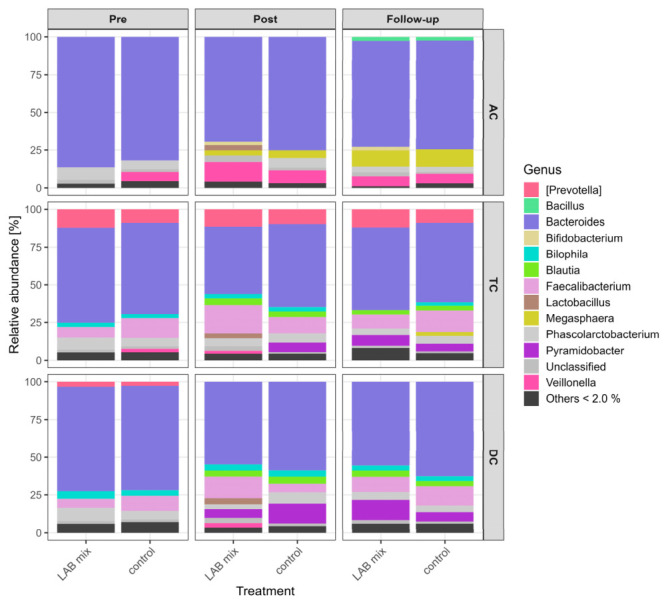
Microbial community changes during the experiment. Relative distribution of major bacterial genera between LAB MIX and control arms across time points (Pre, Post, Follow-up) in three colon segments (AC, TC, DC). Within each panel, stacked bars compare control versus LAB mix groups, with colors denoting each genus. Genera representing < 2.0% of total abundance are grouped as “Others”.

Figure 6 reveals distinct, segment- and time-dependent shifts in the taxonomic composition of the GM at the genus level, using the AC as a baseline because it represents the initial site of colonic fermentation and inoculum stabilization before further regional differentiation occurs in the TC and DC segments. *Bacteroides* emerged as the most dominant genus across all groups, maintaining high relative abundance regardless of the intervention. In the TC and DC (Pre-phase), both the LAB MIX and control arms displayed a uniform distribution, predominantly characterized by *Bacteroides* (~60–70%), with smaller contributions from *Faecalibacterium*, *Phascolarctobacterium*, *Bilophila*, and *Prevotella*. In the post-period time point, the LAB MIX arm demonstrated clear shifts in the TC, characterized by an increase in *Lactobacillus*, *Faecalibacterium*, and *Veillonella* compared to the control arm. Notably, the relative abundance of *Blautia* and *Phascolarctobacterium* showed distinct fluctuations in the LAB-supplemented arm samples, while the control arm maintained a composition closer to the baseline. In the DC, the LAB MIX intervention resulted in similar diversification of the community, evidenced by a relative increase in *Lactobacillus*, which was not observed in the control arm. At the follow-up time point, the taxonomic profile in both the LAB MIX and control arms remained relatively similar to the Post phase across both TC and DC, indicating sustained shifts in microbial community structure. However, the increased relative abundance of the *Lactobacillus* genus observed in the LAB MIX group did not persist into the Follow-up period.

**Figure 7:**
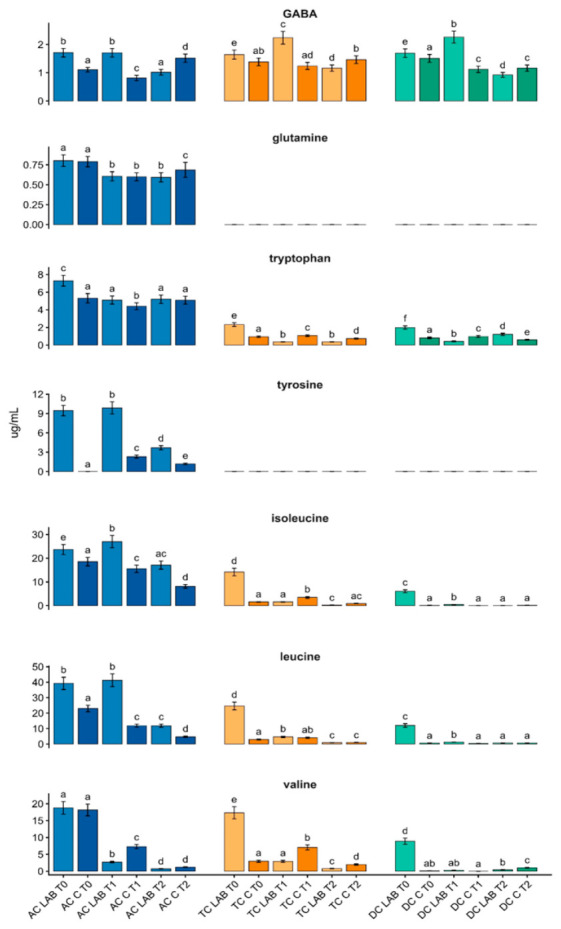
Concentration of selected brain-relevant amino acids between LAB MIX and control (C) arms across time points (Pre—T0, Post—T1, Follow-up—T2) in three colon segments (AC, TC, DC) of the SHIME model. Data are presented as mean values (ug/mL) with standard deviation indicated by error bars. Different lowercase letters (a–f) above the bars denote statistically significant differences between samples for each specific amino acid (one-way ANOVA followed by Tukey’s HSD post hoc test, *p* < 0.05).

To assess the impact of microbial shifts on the metabolic environment, amino acid concentrations were quantified. Specifically, we focused on the concentrations of selected brain-relevant amino acids (Figure 7), while the complete dataset for all measured amino acids is provided in Supplementary Table S1. Supplementation with the MIX significantly increased GABA production (*p* < 0.05), reaching the highest concentrations during the Post-intervention phase, whereas no changes were observed in the control group. Regarding tryptophan, an inverse trend was observed—specifically, a decrease in its concentration in the intestinal contents following MIX administration. 

Concentrations of glutamine and tyrosine dropped below the limit of detection in both arms in Post and Follow-up, which was accompanied by a significant decline in the concentration of all three BCAAs (isoleucine, leucine, valine).

To further investigate the link between microbial shifts and the metabolic environment, PCA was performed to assess the correlations between specific genera and amino acid profiles (Figures S1–S3). In the AC (Figure S1; 72.03% total variance), the LAB mix group (Post and Follow-up) diverged significantly from the control along PC1, correlating with increased *Lactobacillus*, *Faecalibacterium*, *Pyramidobacter*, and *Bilophila*, as well as elevated tyrosine, histidine, and glutamic acid. Furthermore, these changes are characterized by the co-clustering of *Dialister* and *Citrobacter* with a diverse array of metabolic intermediates. Notably, these genera exhibit a strong positive association with BCAAs, such as leucine and isoleucine, as well as a neuroactive metabolic signature comprising key neurotransmitter precursors and mediators, including tryptophan, phenylalanine, and GABA, whereas control samples remained close to the T0 baseline and were associated with *Clostridium*, *Veillonella*, and *Megasphaera*. In the TC (Figure S2; 67.49% total variance) Post and Follow-up phases, the LAB arm showed a longitudinal shift driven by *Lactobacillus*, *Veillonella*, and serine, whereas the control group correlated with *Lachnospira*, *Pyramidobacter*, *Megasphaera*, *Citrobacter*, and *Blautia*, alongside elevated levels of glycine and methionine. Finally, PCA of the DC (Figure S3; 65.26% total variance demonstrated less significant segregation between study arms; only the LAB group in the Post phase (T1) shifted toward positive PC1 loadings, clustering with *Lactobacillus*, *Pyramidobacter*, *Megasphaera*, *Blautia*, and *Lachnospira*, alongside elevated histidine and serine. This group also clustered with *Dialister* and *Oscillospira*, and a neuroactive metabolic module comprising GABA, phenylalanine, and glutamic acid, suggesting a distinct functional link between this microbial community and neurotransmitter-related pathways. LAB samples in Follow-up exhibited a ‘metabolic drift’ back toward the ordination center, suggesting attenuation of the initial intervention-driven reconfiguration and a return to a profile similar to that of the control group and its associated taxa, such as *Veilonella*, *Faecalibacterium*, and *Citrobacter*.

## 4. Discussion

The modulation of the MGBA through psychobiotic interventions represents a promising nutritional strategy for maintaining cognitive performance and attenuating memory decline [19,20]. Fermented foods may serve as integrated interventions that modulate the microbiota through a synergistic blend of beneficial microbes, metabolites, and bioactive compounds [21]. Evidence from both clinical trials and systematic reviews indicates that psychobiotic diets rich in fermented and prebiotic foods represent a novel nutritional approach to targeting the GM in order to enhance mental health and prevent cognitive decline across the lifespan [22,23]. However, due to limited consumption of fermented foods, especially among older adults, a promising strategy is to isolate LAB strains from these foods and develop them into dietary supplements. The current study evaluated the probiotic potential of three food-derived LAB strains (OS4, O24, and M3.3.), both as individual isolates and as a multi-strain consortium (LAB MIX). While the dominance of a single strain can suppress others, making outcomes highly dependent on strain selection, ratios, and substrates, simple consortia may serve as essential models for elucidating interspecies interactions and their collective functional effects [24,25]. Exploring these dynamics is crucial, as accumulating evidence indicates that multi-strain formulations can simultaneously target multiple MGBA pathways, including immune signaling and neurotransmitter mechanisms, often providing broader functional coverage than single strains [26,27,28].

To assess the resistance and survival of the tested strains during transit through the GIT, a static in vitro digestion model was employed. High GI viability is essential for probiotic efficacy, as evidence indicates that clinical effectiveness depends on a sufficient number of viable cells reaching the intestine and remaining viable there [29,30]. In the static digestion model, no significant differences in survival were observed among the tested strains, as all strains exhibited comparable stability, with final concentrations remaining high (>8 log CFU/mL) despite a minor reduction in bacterial counts observed over the course of the experiment. Studies directly comparing the GI viability of multi-strain probiotics with their monoculture counterparts have yielded complex and sometimes inconsistent results. Several studies support the potential advantage of consortia; for instance, a consortium of well-screened LAB isolates demonstrated superior survival under simulated GI conditions compared with individual strains from the same pool [31]. This is further supported by evidence showing that co-culturing two LAB strains yields cells with significantly higher viability after simulated digestive stress and during human consumption than cells produced in monoculture [32]. It should also be emphasized that the LAB MIX used in our study was prepared by combining separately grown bacterial cultures. In other words, the individual strains were grown separately and combined prior to the digestion experiment. This procedure was intended to standardize the proportions of individual strains in the MIX at a 1:1:1 ratio and to prevent inter-strain competition during co-cultivation. It is possible that different preparation and mixing methods reported in the literature contribute to variability in the results. Moreover, a systematic review of 13 clinical studies concluded that survival and persistence are strictly strain- and dose-dependent, and found no consistent advantage for multi-strain systems in gut recovery. These findings suggest that while synergistic effects can enhance robustness, the benefits of a multi-strain matrix are not universal and are highly strain- and combination-specific [33].

Evaluating mucoadhesive capacity was crucial, as the ability to adhere to mucin-type glycoproteins is a key determinant of strain persistence and probiotic functionality within the colon [34,35]. While most in vitro mucin adhesion studies quantify adhesion only within individual strains, our approach allows direct comparison between monocultures and a multi-strain bacterial consortium. In this study, the MIX demonstrated robust adhesive activity, which did not differ significantly from that of the most effective monoculture, M3.3. Although M3.3. achieved the highest individual result, the comparable performance of the MIX suggests that this strain may serve as a strong foundational component, effectively maintaining high adhesive potential even when combined with less active strains such as OS4 and O24. These findings indicate that including less adhesive strains in the MIX does not compromise its overall functional efficacy, suggesting a stable, complementary adhesive profile. On the other hand, other authors have found that synergistic interactions in multi-species biofilms can increase biofilm mass nearly five-fold, supporting the concept of “collective resilience”, in which combined microbes form more stable, protective structures than individual strains [36]. Moreover, multi-strain consortia can strengthen the epithelial barrier and inhibit bacterial translocation more effectively than monocultures, likely due to enhanced adhesion effects that amplify health benefits [37,38].

Beyond survival and colonization, we characterized the functional potential of the LAB strains by comparing amino acid profiles, which highlighted distinct metabolic footprints of the tested strains, with the MIX and M3.3. exhibiting the highest biosynthetic and catabolic activity. PCA revealed that the MIX exhibited the most distinct amino acid profile among the tested variants, placing it farthest from the control medium. The elevated association between the MIX and GABA, the primary inhibitory neurotransmitter, is of particular interest, given the growing evidence that microbial GABA can modulate tight junction proteins and mucins, and inhibit pro-inflammatory NF-κB signaling, supporting the gut barrier and reducing intestinal inflammation [39,40]. Notably, while strain M3.3. showed the closest metabolic affinity to the MIX, only the consortium successfully elevated GABA levels, suggesting an enhanced metabolic effect. The MIX also demonstrated positive correlations with glycine and cysteine, key precursors of glutathione synthesis [41]. Given that the reduced/oxidized glutathione balance is a critical regulator of oxidative stress, cell survival, and signaling in both gut and brain tissues, our findings may suggest a potential antioxidant capacity for the MIX [42,43]. Furthermore, the observed relationship between the MIX and BCAAs aligns with the known metabolic capabilities of LAB, which not only utilize these amino acids during fermentation but also increase their free levels in the medium through extracellular proteolytic activity [44,45]. BCAAs play crucial roles in protein synthesis, energy metabolism, and neurotransmitter production; consequently, their depletion may contribute to synaptic dysfunction and neurodegeneration [46]. The pronounced metabolic divergence observed in the MIX suggests that combining the high-performing M3.3. monoculture with other strains results in metabolic complementarity. This synergy, achieved through the additive effects of diverse bioactive profiles, may substantially enhance the overall antioxidant and metabolic potential of the final formulation.

Building upon these functional observations and our previous study [12], our experiment using the SHIME^®^ model further elucidated the ecological impact of the MIX on the gut microbiota ecosystem derived from an AD patient. This specific microbial background was selected because of the characteristic dysbiosis observed in AD pathogenesis, in which an imbalance in gut taxa disrupts the MGBA through inflammatory, neuroendocrine, and metabolic pathways [47,48]. Crucially, such dysbiosis has been associated with chronic neuroinflammatory processes, hyperactivation of the neuronal immune system, and impaired cognitive functions [49]. We hypothesized that the LAB MIX could serve as a supportive therapeutic strategy by acting as a psychobiotic, potentially facilitating structural rearrangements in the microbiota and metabolic pathways that enhance the functional performance of the MGBA.

It was shown that LAB MIX administration induced a time- and segment-dependent shift in microbiota composition. The intervention significantly enhanced microbial richness and alpha diversity (Chao1, Shannon; *p* < 0.01). Furthermore, dbRDA analysis confirmed that the LAB MIX exerted a statistically significant influence on shaping the community structure, fostering a distinct ecological state compared to the control. However, it should be noted that the treatment grouping accounted for only 8.6% of the total community variance. This indicates that while the consortium’s effect is clear and statistically robust (*p* < 0.01), the vast majority of the variation remains attributable to other dominant factors inherent to the model, such as temporal dynamics and differences between colon segments. In the TC and DC segments, the observed Post-intervention increases in *Lactobacillus*, *Faecalibacterium*, and *Veillonella* suggest that the MIX effectively modulates the microbial community, even though this taxonomic shift—specifically regarding *Lactobacillus*—was transient and did not persist into the Follow-up period. This transience is a common finding in probiotic research, where many LAB and commercial probiotics do not permanently colonize the gut. Instead, they transiently persist for days to weeks while dosing continues or shortly after, yet can still modulate the microbiota and host responses [50,51].

Given the observed shifts in microbial community structure, we further investigated whether these changes altered the culture’s metabolic output. Most of all, by integrating taxonomic and metabolic data through PCA across the AC, TC, and DC segments, we developed a comprehensive functional map that illustrates the interplay between microbial community shifts and the resulting amino acid landscape. Amino acids serve as a vital currency within microbial communities, functioning not only as essential nutrients for species with specialized metabolic requirements but also as key substrates for cross-feeding interactions. In particular, in the distal segments of the gut, where the availability of fermentable fiber diminishes, amino acid fermentation emerges as a favorable metabolic strategy. This shift likely promotes mutualistic interactions among diverse microbial species, thereby reinforcing the ecosystem’s resilience [52]. Crucially, in vitro studies suggest that such cross-feeding serves as an ecological buffer, stabilizing the microbial community against dietary perturbations [53]. The clear divergence between the treatment and control arms, particularly in the AC and TC arms, underscores the consortium’s capacity to actively steer the microbiota toward a distinct metabolic state, rather than merely increasing the abundance of individual taxa. These metabolic shifts were organized into three distinct functional modules, linking the reconfigured microbiota to key neuromodulatory pathways. Specifically, in the LAB MIX arm, we noted a catecholaminergic module [52] comprising tyrosine and phenylalanine and a GABAergic module [53] comprising glutamic acid and GABA, which strongly co-cluster with *Lactobacillus*, *Faecalibacterium*, *Dialister*, and *Citrobacter*. Furthermore, depletion of tryptophan—the precursor to serotonin [54], and enrichment in histidine—the precursor to histamine, which may serve as a neurotransmitter [55]—were observed. While tryptophan depletion may indicate conversion to serotonin or neuroprotective indoles, it may also limit availability for the host’s kynurenine pathway, the primary route for NAD+ synthesis. Although the role of the kynurenine pathway remains incompletely understood and its link to inflammation is inconsistent across studies, its dysregulation can produce neurotoxic metabolites linked to AD, suggesting that microbial utilization of this amino acid may significantly influence host neuro-immune homeostasis [56]. The study also revealed elevated serine levels, which correlated mostly with *Lactobacillus*, *Veillonella*, and *Pyramidobacter.* Serine is vital for protein synthesis, neurotransmission, and lipid metabolism; moreover, by regulating NF-κB signaling and mitigating oxidative stress, it may support gut microbial homeostasis [57,58]. Some studies suggest that increasing luminal BCAA levels *via* probiotics can be neuroprotective [59] and that plasma levels of glutamine, BCAAs, serine, methionine, and phenylalanine influence neurotransmission by serving as direct precursors for glutamate, aspartate, and glycine synthesis, as well as by modulating the competitive transport of tryptophan and tyrosine across the blood–brain barrier [60]. On the other hand, several studies have demonstrated that reduced BCAA levels are associated with AD-related pathology and cognitive deficits [61,62]. Our findings demonstrate a significant decline in BCAA levels, while tyrosine and glutamine concentrations dropped below the limit of detection in the distal colon compartments (TC and DC). As the medium flows sequentially through the system, this regional drop reflects a highly active fermentation state and intensive microbial catabolism in the subsequent sections of the large intestine, which may be considered a donor-specific microbiota feature. Future research should clarify whether this intensive microbial consumption limits the systemic availability of these precursors for the host or represents a necessary trade-off for the production of potent neuroactive compounds.

While these results suggest robust potential for metabolic cross-feeding, they have several limitations and must be interpreted within the context of the model’s architecture. Unlike the human GI tract, where the host epithelium actively absorbs luminal metabolites, dynamic in vitro models such as the SHIME^®^ lack the complexity of the human organism, including its absorption and secretory functions [63]. Consequently, the elevated amino acid levels reflect a net balance between microbial production and consumption rather than in vivo steady-state concentrations. Although this ‘accumulation effect’ magnifies metabolic interactions that are typically masked by host absorption, the SHIME^®^ model provides valuable insights into the consortium’s metabolic and functional potential. Given that the initial evaluation relied on a single donor, our findings should be considered exploratory, case-study observations rather than definitive, generalizable results. To capture inter-individual variability and enable robust statistical validation, these findings must be confirmed in future independent trials involving microbiota from multiple donors. Furthermore, while capturing fully stabilized metabolic steady states at the conclusion of each phase was crucial, future studies could further expand on these insights by incorporating shorter time-interval sampling to map the underlying temporal dynamics more precisely. While 16S rRNA Illumina sequencing effectively mapped broader taxonomic shifts, its shorter read lengths limited strain resolution within *Lactobacillus* sensu lato. To accurately distinguish the exogenous MIX strains from the native background microbiota, future studies should incorporate high-resolution approaches, such as strain-specific qPCR, shotgun metagenomics, or long-read sequencing technologies, to precisely monitor strain survival and colonization kinetics. Finally, further in vivo studies and randomized clinical trials are required to confirm these effects in patients.

In summary, our findings demonstrate that LAB MIX acts as a potent metabolic catalyst, actively fostering a more resilient, metabolically dynamic ecosystem. Beyond promoting microbial growth, these interactions can generate a range of neuroactive and antioxidant metabolites with significant health-modulatory potential.

## 5. Conclusions

Our study demonstrates that the food-derived multi-strain LAB consortium (LAB MIX) exhibits a robust functional profile, characterized by high survival rates during simulated gastrointestinal transit, effective mucoadhesive properties, and a distinct metabolic signature, which suggests a mild complementary effect that exceeds the functional potential of the individual monocultures. Furthermore, our results suggest that the multi-strain MIX acts as a metabolic modulator, driving significant shifts in neuroactive and antioxidant amino acid profiles, characterized by a modest elevation in GABA alongside the intensive utilization of other key precursors, within a dynamic SHIME model inoculated with microbiota from an Alzheimer’s disease patient. These findings highlight the consortium’s psychobiotic potential and its capacity to influence pathways relevant to the microbiota–gut–brain axis. While these in vitro results provide foundational evidence, further studies are required to validate these findings, as the in vitro SHIME model, while effectively demonstrating microbial-driven metabolic mechanisms, lacks the complexity of the host-absorptive systemic environment. Future studies should aim to expand this metabolic profile by investigating additional pathways, such as SCFA production, to fully capture the broader spectrum of gut microbial activities, as well as to determine whether this ‘metabolic signature’ is sufficient to translate into systemic host benefits and to establish the optimal frequency of intervention required to maintain this modulated state. Consequently, these results must be confirmed through in vivo and clinical investigations to fully elucidate the physiological impact of the LAB MIX on the MGBA in humans.

## Figures and Tables

**Table 1:** The experimental procedure for each phase of the simulated in vitro digestion.

Digestion Phase	Digestion Procedure
**Oral Phase**	The bacterial suspension (strains OS4, O24, M3.3, and LAB MIX) was mixed with the corresponding SSF in a 1:1 ratio (100 µL:100 µL). The sample was incubated for 2 min at 37 °C with continuous stirring.
**Gastric Phase**	The fluid obtained following the oral phase was mixed with the corresponding SGF in a 1:1 ratio (200 µL:200 µL). The sample was incubated for 2 h at 37 °C with continuous stirring.
**Intestinal Phase**	The fluid obtained following the gastric phase was mixed with the corresponding SIF in a 1:1 ratio (400 µL:400 µL). The sample was incubated for 2 h at 37 °C with continuous stirring.

## Data Availability

The data supporting this study’s findings are openly available in the RepOD repository under DOI: 10.18150/NEGFZD (https://doi.org/10.18150/NEGFZD).

## Supplementary Materials

The following supporting information can be downloaded at: https://www.mdpi.com/article/10.3390/nu18121946/s1, Figure S1: PCA biplot of the AC segment based on 16S rRNA genus profiles and free amino acids, comparing the LAB and control (C) experimental arms across three study time points: Pre/baseline (T0), Post intervention (T1), and Follow-up (T2); Figure S2: PCA biplot of the TC segment based on 16S rRNA genus profiles and free amino acids, comparing the LAB and control (C) experimental arms across three study time points: Pre/baseline (T0), Post intervention (T1), and Follow-up (T2); Figure S3: PCA biplot of the DC segment based on 16S rRNA genus profiles and free amino acids, comparing the LAB and control (C) experimental arms across three study time points: Pre/baseline (T0), Post intervention (T1), and Follow-up (T2); Table S1: Concentration of amino acids in LAB MIX and control (C) arms across time points (Pre—T0, Post—T1, Follow-up—T2) in three colon segments (AC, TC, DC) of the SHIME model. Data are presented as mean values (ug/mL) ± standard deviation.

## Abbreviations

The following abbreviations are used in this manuscript:

AC	Ascending colon
AD	Alzheimer’s disease
BCAAs	Branched-chain amino acids
CFU	Colony-forming units
DC	Descending colon
MGBA	Microbiota–gut–brain axis
GIT	Gastrointestinal tract
GM	Gut microbiota
LAB	Lactic acid bacteria
SCFAs	Short-chain fatty acids
SHIME	Simulator of the Human Intestinal Microbial Ecosystem
TC	Transverse colon
