# Supplementary material for: Functional and Psychobiotic Potential of a Food-Derived Multi-Strain Lactic Acid Bacteria Consortium: An In Vitro Evaluation Using Static Digestion and SHIME® Models
Source: Nutrients. 2026 Jun 16;18(12):1946. doi: 10.3390/nu18121946 (PMC13305391; doi:10.3390/nu18121946)
Supplement: Supplementary file 1 [file nutrients-18-01946-s001.zip › nutrients-4348567-supplementary.pdf]

Supplementary material

Synergistic Effect of Food-Derived Lactic Acid Bacteria - A Multi-Strain Consortium for Potential Psychobiotic Applications

Wioletta Mosiej, Marcin Kruk, Tomasz Królikowski, Michał Oczkowski, Klaudia Glegoła, Dorota Zielińska*

Table S1. Concentration of amino acids in LAB MIX and control (C) arms across time points (Pre - T0, Post - T1, Follow-up - T2) in three colon segments (AC, TC, DC) of the SHIME model. Data are presented as mean values (ug/mL) ± standard deviation.

Amino acid / Sample	AC LAB T0	AC C T0	AC LAB T1	AC C T1	AC LAB T2	AC C T2	TC LAB T0	TC C T0	TC LAB T1	TC C T1	TC LAB T2	TC C T2	DC LAB T0	DC C T0	DC LAB T1	DC C T1	DC LAB T2	DC C T2
**Aspartic acid**	15,56 ± 0,32	11,85 ± 0,35	13,61 ± 0,37	4,11 ± 0,12	18,11 ± 0,60	2,50 ± 0,07	2,00 ± 0,07	1,68 ± 0,06	0,94 ± 0,03	1,18 ± 0,04	0,40 ± 0,02	1,12 ± 0,04	0,89 ± 0,03	0,19 ± 0,01	0,14 ± 0,01	0,00 ± 0,00	0,00 ± 0,00	0,00 ± 0,00
**Serine**	16,97 ± 0,51	7,79 ± 0,19	12,62 ± 0,34	2,19 ± 0,06	1,59 ± 0,05	1,59 ± 0,05	0,72 ± 0,02	0,80 ± 0,02	0,90 ± 0,02	0,88 ± 0,02	0,90 ± 0,02	0,78 ± 0,02	0,71 ± 0,02	0,69 ± 0,02	0,78 ± 0,02	0,78 ± 0,02	0,82 ± 0,02	0,72 ± 0,02
**Glycine**	26,46 ± 0,70	37,42 ± 1,16	31,63 ± 0,99	31,68 ± 0,99	38,07 ± 1,19	34,67 ± 1,14	0,76 ± 0,02	0,58 ± 0,02	0,46 ± 0,02	1,20 ± 0,04	0,70 ± 0,02	2,56 ± 0,08	0,49 ± 0,02	0,50 ± 0,02	0,46 ± 0,02	0,30 ± 0,01	0,24 ± 0,01	0,24 ± 0,01
**GABA**	1,71 ± 0,05	1,11 ± 0,03	1,71 ± 0,05	0,81 ± 0,03	1,02 ± 0,03	1,52 ± 0,05	1,64 ± 0,05	1,38 ± 0,04	2,24 ± 0,07	1,24 ± 0,04	1,16 ± 0,04	1,46 ± 0,04	1,69 ± 0,05	1,51 ± 0,05	2,26 ± 0,07	1,12 ± 0,04	0,92 ± 0,03	1,16 ± 0,04
**Arginine**	20,71 ± 0,60	23,77 ± 0,69	17,49 ± 0,56	20,56 ± 0,63	22,69 ± 0,71	20,36 ± 0,65	8,08 ± 0,27	2,42 ± 0,08	0,70 ± 0,02	6,64 ± 0,22	0,46 ± 0,02	7,68 ± 0,25	1,00 ± 0,03	0,50 ± 0,02	0,60 ± 0,02	0,20 ± 0,01	0,38 ± 0,01	1,48 ± 0,05
**Tyrosine**	9,46 ± 0,27	0,00 ± 0,00	9,87 ± 0,31	2,31 ± 0,08	3,69 ± 0,11	1,19 ± 0,04	0,00 ± 0,00	0,00 ± 0,00	0,00 ± 0,00	0,00 ± 0,00	0,00 ± 0,00	0,00 ± 0,00	0,00 ± 0,00	0,00 ± 0,00	0,00 ± 0,00	0,00 ± 0,00	0,00 ± 0,00	0,00 ± 0,00
**Cysteine**	25,17 ± 0,68	24,34 ± 0,65	4,19 ± 0,12	10,09 ± 0,28	1,49 ± 0,04	2,11 ± 0,06	23,29 ± 0,77	4,45 ± 0,14	4,40 ± 0,15	9,82 ± 0,32	1,54 ± 0,05	3,17 ± 0,10	12,19 ± 0,41	0,70 ± 0,02	0,92 ± 0,03	0,28 ± 0,01	1,10 ± 0,03	1,90 ± 0,06
**Methionine**	6,30 ± 0,19	5,92 ± 0,17	6,29 ± 0,19	3,31 ± 0,09	6,40 ± 0,20	4,28 ± 0,14	0,60 ± 0,02	0,90 ± 0,03	0,60 ± 0,02	2,72 ± 0,09	0,52 ± 0,02	1,14 ± 0,03	0,41 ± 0,02	0,59 ± 0,02	0,54 ± 0,02	0,26 ± 0,01	0,36 ± 0,01	0,92 ± 0,03
**Tryptophan**	7,30 ± 0,20	5,31 ± 0,18	5,11 ± 0,15	4,40 ± 0,13	5,21 ± 0,16	5,09 ± 0,15	2,32 ± 0,07	0,94 ± 0,03	0,36 ± 0,01	1,08 ± 0,03	0,36 ± 0,01	0,74 ± 0,02	1,99 ± 0,06	0,81 ± 0,03	0,42 ± 0,02	0,98 ± 0,03	1,22 ± 0,04	0,60 ± 0,02
**Lysine**	19,64 ± 0,57	32,26 ± 0,94	29,22 ± 0,98	27,56 ± 0,84	33,35 ± 1,05	24,90 ± 0,83	2,60 ± 0,08	2,26 ± 0,07	1,38 ± 0,04	1,22 ± 0,04	1,40 ± 0,05	0,88 ± 0,02	2,20 ± 0,07	1,90 ± 0,06	1,28 ± 0,04	1,08 ± 0,03	0,98 ± 0,03	0,92 ± 0,03
**Glutamic acid**	38,72 ± 1,17	42,27 ± 1,34	40,58 ± 1,27	37,96 ± 1,20	46,75 ± 1,70	38,84 ± 1,25	3,14 ± 0,10	1,98 ± 0,06	2,52 ± 0,09	1,72 ± 0,06	1,92 ± 0,07	1,88 ± 0,07	1,80 ± 0,06	1,31 ± 0,04	2,24 ± 0,07	1,06 ± 0,03	1,52 ± 0,05	1,26 ± 0,04
**Asparagine**	0,39 ± 0,01	0,40 ± 0,02	0,00 ± 0,00	0,71 ± 0,03	0,39 ± 0,01	0,70 ± 0,02	0,00 ± 0,00	0,00 ± 0,00	0,00 ± 0,00	0,00 ± 0,00	0,00 ± 0,00	0,00 ± 0,00	0,00 ± 0,00	0,00 ± 0,00	0,00 ± 0,00	0,00 ± 0,00	0,00 ± 0,00	0,00 ± 0,00
**Glutamine**	0,80 ± 0,02	0,79 ± 0,02	0,61 ± 0,02	0,60 ± 0,02	0,59 ± 0,02	0,69 ± 0,03	0,00 ± 0,00	0,00 ± 0,00	0,00 ± 0,00	0,00 ± 0,00	0,00 ± 0,00	0,00 ± 0,00	0,00 ± 0,00	0,00 ± 0,00	0,00 ± 0,00	0,00 ± 0,00	0,00 ± 0,00	0,00 ± 0,00
**Histidine**	8,89 ± 0,28	11,11 ± 0,31	11,93 ± 0,33	10,40 ± 0,31	11,93 ± 0,33	9,50 ± 0,30	0,04 ± 0,00	0,12 ± 0,00	0,06 ± 0,00	0,06 ± 0,00	0,06 ± 0,00	0,04 ± 0,00	0,10 ± 0,00	0,10 ± 0,00	0,28 ± 0,01	0,18 ± 0,01	0,14 ± 0,01	0,10 ± 0,00
**Threonine**	17,52 ± 0,57	9,06 ± 0,26	28,63 ± 0,97	2,20 ± 0,06	13,09 ± 0,41	1,22 ± 0,04	1,46 ± 0,05	2,14 ± 0,07	0,70 ± 0,02	0,68 ± 0,02	0,58 ± 0,02	0,60 ± 0,02	0,71 ± 0,02	0,71 ± 0,02	0,56 ± 0,02	0,42 ± 0,02	0,26 ± 0,01	0,26 ± 0,01
**Alanine**	10,74 ± 0,31	31,42 ± 0,88	23,22 ± 0,76	40,77 ± 1,33	16,23 ± 0,52	34,08 ± 1,12	5,70 ± 0,19	4,68 ± 0,16	0,18 ± 0,01	5,24 ± 0,18	0,00 ± 0,00	6,12 ± 0,21	1,39 ± 0,05	0,30 ± 0,01	0,64 ± 0,02	0,00 ± 0,00	0,46 ± 0,02	0,48 ± 0,02
**Proline**	15,22 ± 0,43	5,71 ± 0,18	5,71 ± 0,18	3,18 ± 0,10	1,10 ± 0,03	0,69 ± 0,03	0,00 ± 0,00	0,00 ± 0,00	0,00 ± 0,00	0,00 ± 0,00	0,00 ± 0,00	0,00 ± 0,00	0,00 ± 0,00	0,00 ± 0,00	0,00 ± 0,00	0,00 ± 0,00	0,00 ± 0,00	0,00 ± 0,00
**Valine**	18,79 ± 0,61	18,16 ± 0,58	2,71 ± 0,08	7,29 ± 0,20	0,71 ± 0,02	1,20 ± 0,04	17,32 ± 0,59	2,96 ± 0,10	2,92 ± 0,10	7,06 ± 0,24	0,76 ± 0,02	1,98 ± 0,07	8,90 ± 0,30	0,11 ± 0,01	0,28 ± 0,01	0,00 ± 0,00	0,42 ± 0,02	1,02 ± 0,03
**Isoleucine**	23,67 ± 0,69	18,56 ± 0,59	27,00 ± 0,85	15,54 ± 0,49	17,10 ± 0,57	8,12 ± 0,26	14,19 ± 0,52	1,56 ± 0,05	1,56 ± 0,05	3,44 ± 0,11	0,24 ± 0,01	0,90 ± 0,02	6,09 ± 0,19	0,09 ± 0,02	0,44 ± 0,02	0,00 ± 0,00	0,00 ± 0,00	0,14 ± 0,01
**Leucine**	39,25 ± 1,31	23,00 ± 0,70	41,24 ± 1,36	11,89 ± 0,30	11,83 ± 0,33	4,70 ± 0,15	24,64 ± 0,82	2,94 ± 0,10	4,64 ± 0,15	4,00 ± 0,13	0,88 ± 0,02	0,98 ± 0,03	12,00 ± 0,38	0,61 ± 0,02	1,22 ± 0,03	0,40 ± 0,01	0,64 ± 0,02	0,62 ± 0,02
**Phenylalanine**	22,91 ± 0,78	10,68 ± 0,28	22,95 ± 0,71	5,01 ± 0,12	6,22 ± 0,18	1,90 ± 0,06	16,75 ± 0,56	3,84 ± 0,13	1,00 ± 0,03	3,36 ± 0,11	0,28 ± 0,01	0,96 ± 0,03	0,31 ± 0,01	0,00 ± 0,00	0,34 ± 0,01	0,00 ± 0,00	0,00 ± 0,00	0,28 ± 0,01

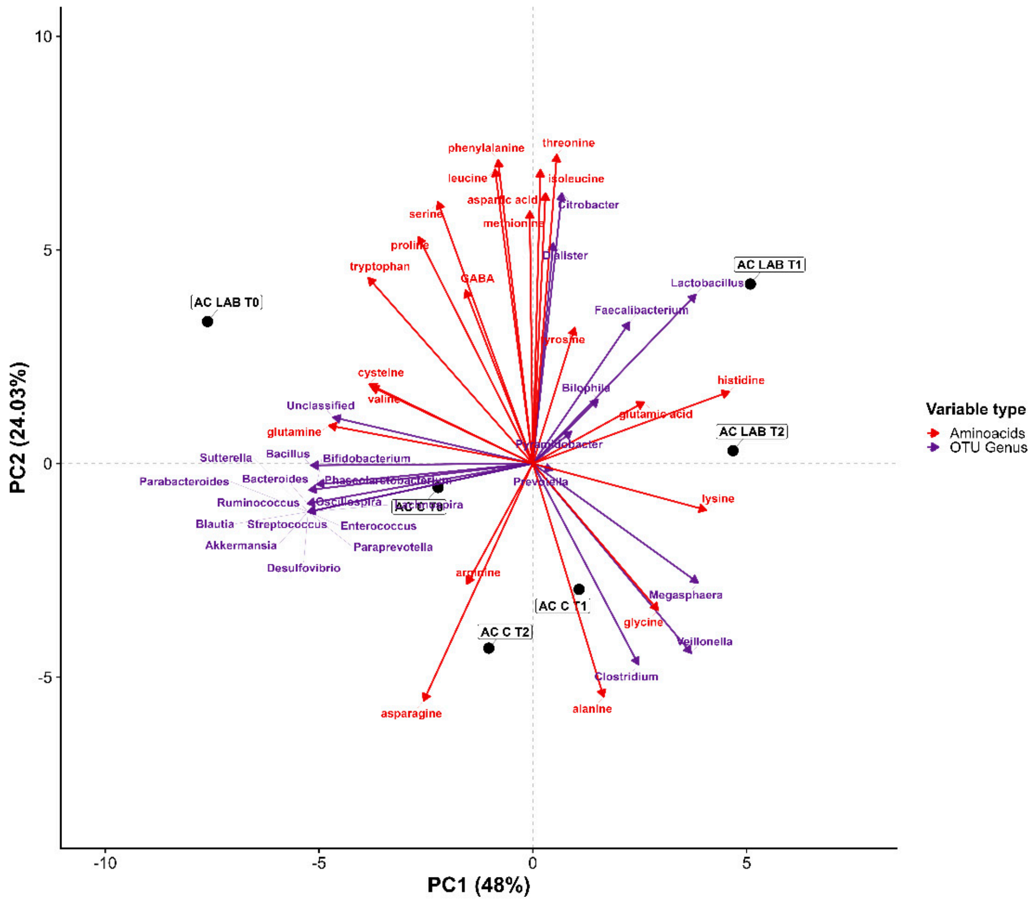

Figure S1. PCA biplot of the AC segment based on 16S rRNA genus profiles and free amino acids, comparing the LAB and control (C) experimental arms across three study time points: Pre/baseline (T0), Post intervention (T1), and Follow-up (T2). Points represent individual samples, and arrows represent variables. The direction and length of the arrows indicate their contribution to the principal components, while the projection of samples onto the arrows reflects the correlation between variables and samples. The percentage of variance explained by each principal component is indicated on the axes.

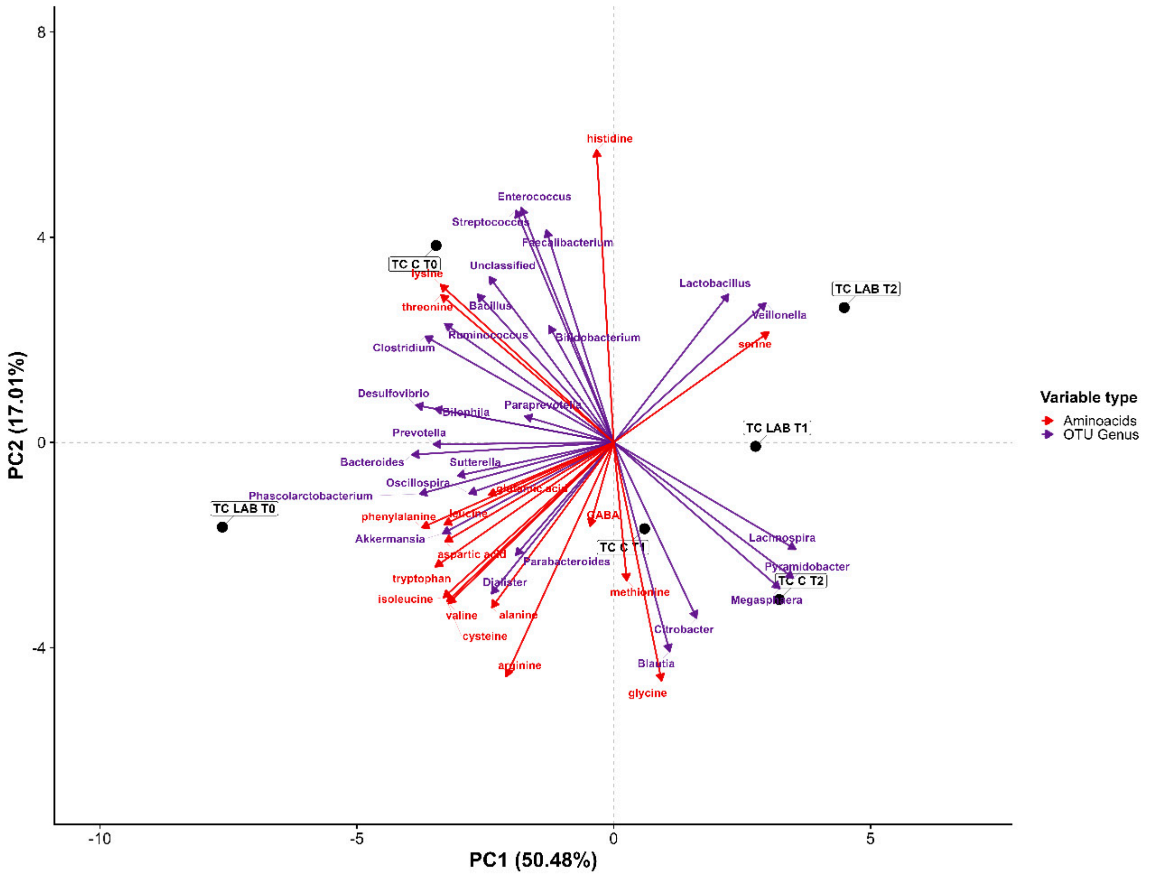

Figure S2. PCA biplot of the TC segment based on 16S rRNA genus profiles and free amino acids, comparing the LAB and control (C) experimental arms across three study time points: Pre/baseline (T0), Post intervention (T1), and Follow-up (T2). Points represent individual samples, and arrows represent variables. The direction and length of the arrows indicate their contribution to the principal components, while the projection of samples onto the arrows reflects the correlation between variables and samples. The percentage of variance explained by each principal component is indicated on the axes.

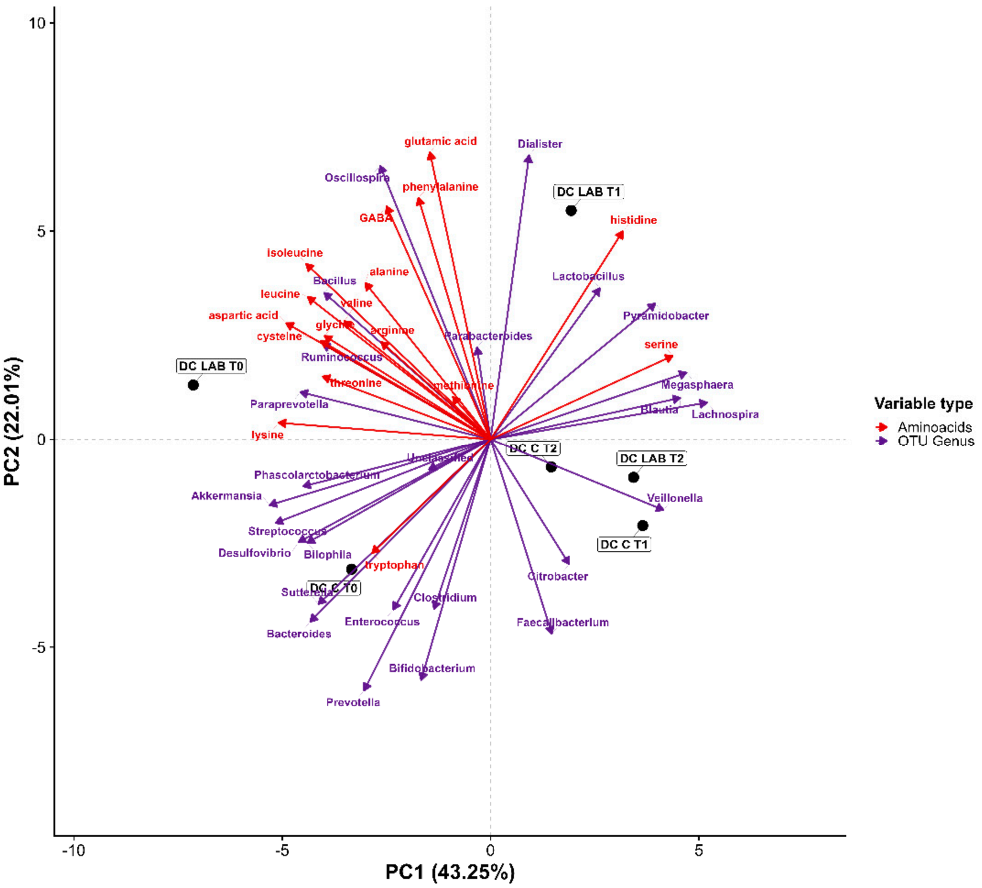

Figure S3. PCA biplot of the DC segment based on 16S rRNA genus profiles and free amino acids, comparing the LAB and control (C) experimental arms across three study time points: Pre/baseline (T0), Post intervention (T1), and Follow-up (T2). Points represent individual samples, and arrows represent variables. The direction and length of the arrows indicate their contribution to the principal components, while the projection of samples onto the arrows reflects the correlation between variables and samples. The percentage of variance explained by each principal component is indicated on the axes.
